# The Development of a Portable Hard Disk Encryption/Decryption System with a MEMS Coded Lock

**DOI:** 10.3390/s91109300

**Published:** 2009-11-19

**Authors:** Weiping Zhang, Wenyuan Chen, Jian Tang, Peng Xu, Yibin Li, Shengyong Li

**Affiliations:** Key Laboratory for Thin Film and Microfabrication of Ministry of Education, National Key Laboratory of Nano/Micro Fabrication Technology, Research Institute of Micro/Nano Technology, Shanghai Jiao Tong University, Shanghai 200240, China; E-Mails: chenwy@sjtu.edu.cn (W.C.); SJTUtj@126.com (J.T.); xpsjtu@sohu.com (P.X.); ybsjtu@sohu.com (Y.L.); lshyong@tom.com (S.L.)

**Keywords:** portable hard disk encryption/decryption system, MEMS coded lock, FPGA

## Abstract

In this paper, a novel portable hard-disk encryption/decryption system with a MEMS coded lock is presented, which can authenticate the user and provide the key for the AES encryption/decryption module. The portable hard-disk encryption/decryption system is composed of the authentication module, the USB portable hard-disk interface card, the ATA protocol command decoder module, the data encryption/decryption module, the cipher key management module, the MEMS coded lock controlling circuit module, the MEMS coded lock and the hard disk. The ATA protocol circuit, the MEMS control circuit and AES encryption/decryption circuit are designed and realized by FPGA(Field Programmable Gate Array). The MEMS coded lock with two couplers and two groups of counter-meshing-gears (CMGs) are fabricated by a LIGA-like process and precision engineering method. The whole prototype was fabricated and tested. The test results show that the user's password could be correctly discriminated by the MEMS coded lock, and the AES encryption module could get the key from the MEMS coded lock. Moreover, the data in the hard-disk could be encrypted or decrypted, and the read-write speed of the dataflow could reach 17 MB/s in Ultra DMA mode.

## 1. Introduction

Various security threats, such as the malicious data modification, data leaks and the stolen hard-disk events, may cause inestimable loss to some organizations, such as the military, governments and enterprises. As more and more important data is stored in portable hard disks, how to protect the data has become a hot issue. There are mainly two encryption approaches to protect the data in the portable hard disk: the software-based method and the hardware-based method.

The software-based method uses the computer's CPU to perform encryption/decryption tasks. But this kind of method has some disadvantages: (1) The encryption/decryption software can easily to be monitored by a Trojan program; (2) The instructions of the encryption/decryption are executed by the CPU, which will consume more computer resources; (3) It is difficult to transfer the encryption/decryption software among different operating systems. The K210 portable hard disk of Netac Technology Company, NetDisk Mini portable hard disk of Ximeta Company and Truecrypt Foundation use the software encrypted/decryption method [1-4].

The hardware-based method uses special chips to accelerate the encryption/decryption process. This method needs less computer resources. The E906 portable hard-disk of the EAGET company, the P681 portable hard-disk of the Agio company and the Drive trust hard-disk of Seagate company use the hardware encryption/decryption method [5-7].

Usually, the key is stored in special sectors of the hard disk or USB's flash in both of software-based and the hardware-based encryption/decryption system. The security of hardware-based hard-disk encryption/decryption is higher than that of the software-based hard-disk encryption/decryption. However, it is still possible that the key can be broken because the key is usually stored in special sectors of the hard-disk or USB's flash.

In this paper, a MEMS coded lock is added in the hardware-based encryption/decryption system. The user's password is discriminated by the MEMS coded lock, and then the key stored in the MEMS coded lock is transferred into the AES encryption/decryption module. It is very difficult to break the key from the mechanical maze (two groups of CMGs), thus the security of the hardware-based encryption/decryption system with a MEMS coded lock is greatly increased. The paper is arranged as follows: In Section 2, the framework of the portable hard disk encryption/decryption system is described. In Section 3, the design of the USB interface card is introduced. In Section 4, the ATA protocol command decoder module is given. In Section 5, the data hardware encryption/decryption is studied. In Section 6, the controlling circuit of the MEMS coded lock is studied. At last, this paper gives the tested results of the first generation prototype of the portable hard disk encryption/decryption system with MEMS coded lock.

## 2. The Frame Work of the Portable Hard Disk Encryption/Decryption System with MEMS Coded Lock

### 2.1. The Structure of the Portable Hard Disk Encryption/Decryption System

**Figure 1.**
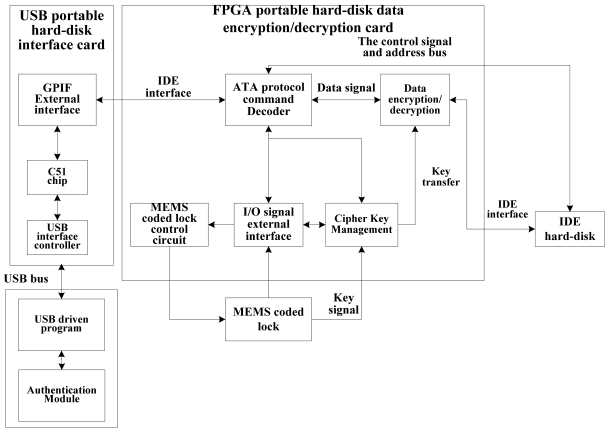
The frame work of the portable hard disk encryption/decryption system with MEMS coded lock.

Figure 1 shows the portable hard disk encryption/decryption system with MEMS coded lock, which is realized by FPGA.

The system includes a USB portable hard disk interface card, a FPGA portable hard disk data encryption/decryption card, an authentication module, a MEMS coded lock and a hard disk. The USB portable hard disk interface card is composed of a GPIF's external interface module, a C51 chip and a USB interface controller. The FPGA portable hard disk data encryption/decryption card consists of an ATA protocol command decoder, a data encryption/decryption module, an I/O external interface, a MEMS coded lock circuit and a cipher key management module. The FPGA portable hard disk data encryption/decryption card can be regarded as the ATA protocol storage device from the perspective of the host computer. The FPGA portable hard disk data encryption/decryption card can also be regarded as the ATA protocol host controller from the perspective of the hard disk. The IDE interface circuit and encryption/decryption circuit are realized by FPGA method.

### 2.2. The Two Important Functions of the Portable Hard Disk Encryption/Decryption System

#### 2.2.1. The main functions relative to the MEMS coded lock

The MEMS coded lock has a mechanical maze to store the mechanical key. The main functions relative to the MEMS coded lock are to authenticate a user's password and provide the cipher key for the encryption/decryption module by means of the authentication module, the MEMS coded lock control circuit and the cipher key management module.

**Figure 2.**
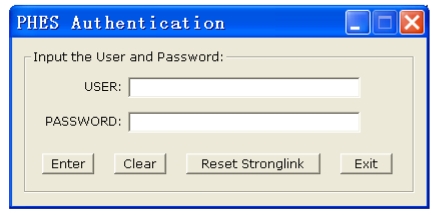
The interface of the authentication module.

As indicated in Figure 1 and Figure 2, the authentication module transforms the entered password into the driving instructions of the MEMS coded lock. Then these instructions are sent to the MEMS coded lock control circuit module. Thus, the password is converted to a mechanical movement of the MEMS coded lock. If the user's password is correct, the mechanical maze will be passed. Otherwise, the mechanical structure will be locked up. The cipher key management module judges whether the MEMS coded lock is locked up or not according to the feedback signal of the MEMS coded lock. If the user's password has matched with the key stored in the mechanical structure of the MEMS coded lock, the cipher key management module will send a signal to inform the host computer that the user's password is correct, and transfers the key to the AES encryption/decryption module. If the user's password is not matched with the key stored in the mechanical structure of the MEMS coded lock, the key management module will send the authentication failure signal to the host computer, and the host computer will reset the MEMS coded lock.

#### 2.2.2. The main functions relative to the data encryption/decryption

**Figure 3.**
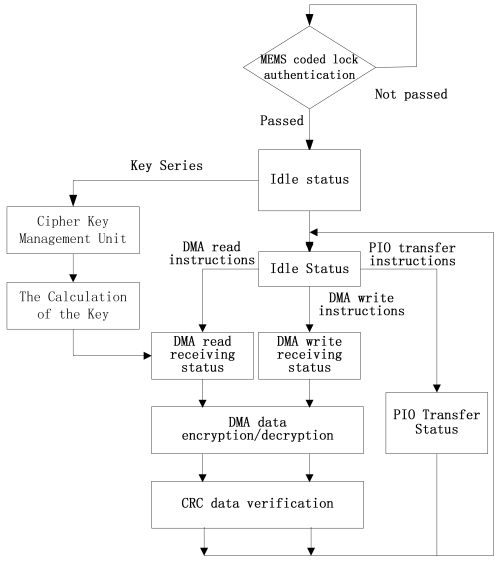
The signal transmission flow of the portable hard disk encryption/decryption system.

The functions relative to the data encryption/decryption are the AES arithmetic circuit and the data's transfer between the host computer and the hard disk. The AES arithmetic circuit is realized by FPGA. The data is transferred by the UDMA and PIO channel. The FPGA portable hard-disk data encryption/decryption card (see Figure 1) receives the IDE instructions from the GPIF external interface. The signal flow is shown in Figure 3. The UDMA channel or the PIO channel is selected according to the analyses of ATA protocol instructions. In the UDMA channel, the data are encrypted or decrypted using the key from the MEMS coded lock. In PIO channel, the data are not changed. The details of the UDMA/PIO channel will be discussed in the following sections.

## 3. USB Interface Controller

USB interface controller is the bridge between the FPGA portable hard-disk data encryption/decryption card and the host computer. The USB interface controller adopts EZ-USB FX2 of Cypress [8]. In the USB interface controller, GPIF implements ATA protocols, such as the PIO protocol and the UDMA protocol.

### 3.1. The Work Flow of the USB Interface Controller

When the portable hard-disk encryption system is plugged into the host computer, EZ-USB FX2 enumerates automatically and downloads firmware and USB descriptor tables. The host computer will identify EZ-USB FX2 as the development board of EZ-USB FX2. Then EZ-USB FX2 enumerates again as EZ-USB FX2 sample device. If the user passes the authentication of the MEMS coded lock, EZ-USB FX2 enumerates again as the hard disk. If the user does not pass the authentication of the MEMS coded lock, the hard disk cannot be renumerated, so the host computer cannot identify the hard disk.

### 3.2. The Design of the GPIF's Waveform

**Figure 4.**
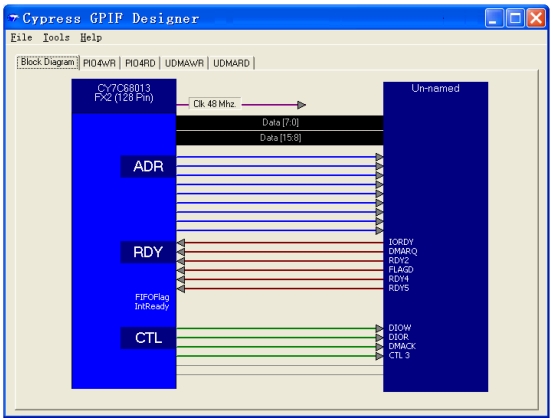
Interface Definition of GPIF for ATAPI.

**Figure 5.**
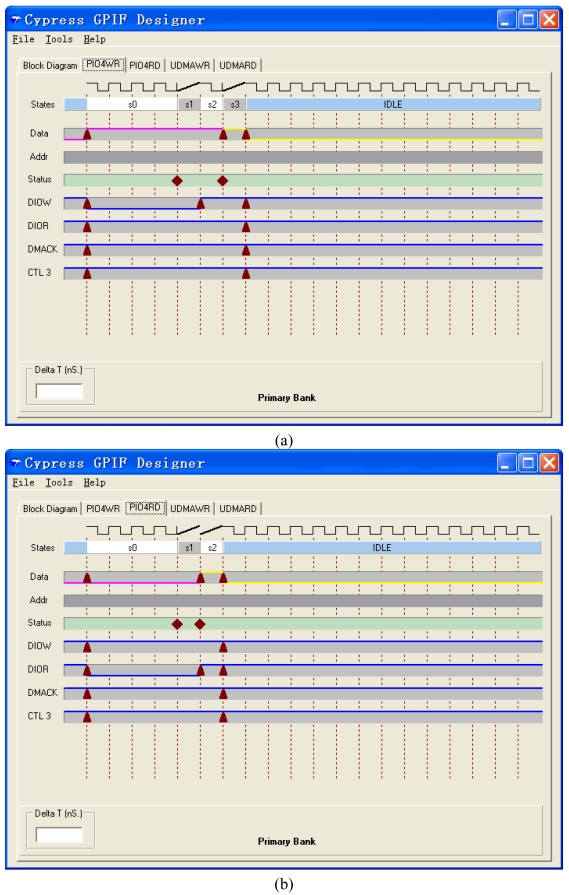
Waveform Descriptions of (a) PIOWR and (b) PIORD.

**Figure 6.**
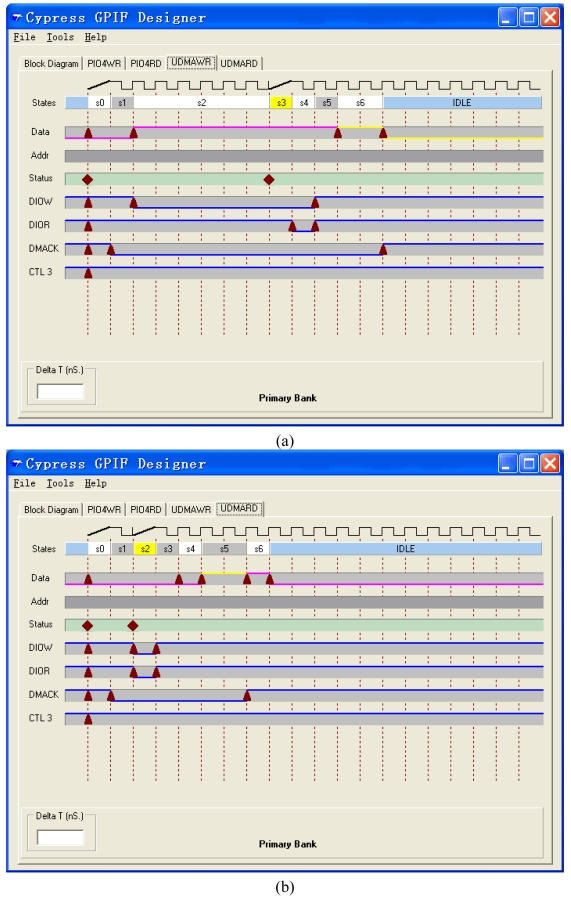
Waveform Descriptions of (a) UDMAWR and (b) UDMARD.

The ATAPI interface is realized by GPIF, whose waveform is designed by the GPIF software of Cypress (Figure 4). The data bus is 16 bits width. The clock frequency of the interface is 48 MHz. The address bus is 9 bits width. The three control output pins are DIOW (the IO writing signal), DIOR (the IO reading signal) and DMACK (the DMA acknowledgement signal). The three input pins are IORDY (the IO ready signal), DMARQ (the DMA acknowledgement signal) and FLAGD (the flag signal). Four waveform descriptors are defined as PIOWR, PIORD, UDMARD and UDMAWR and shown in Figures 5 and 6.

## 4. The ATA Protocol Command Decoder Module

**Figure 7.**
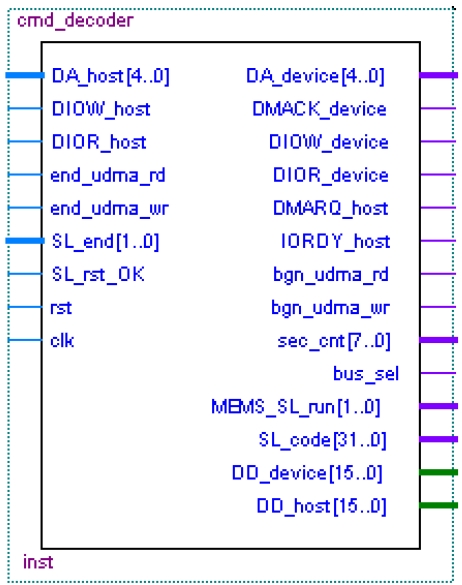
Command decode module.

**Figure 8.**
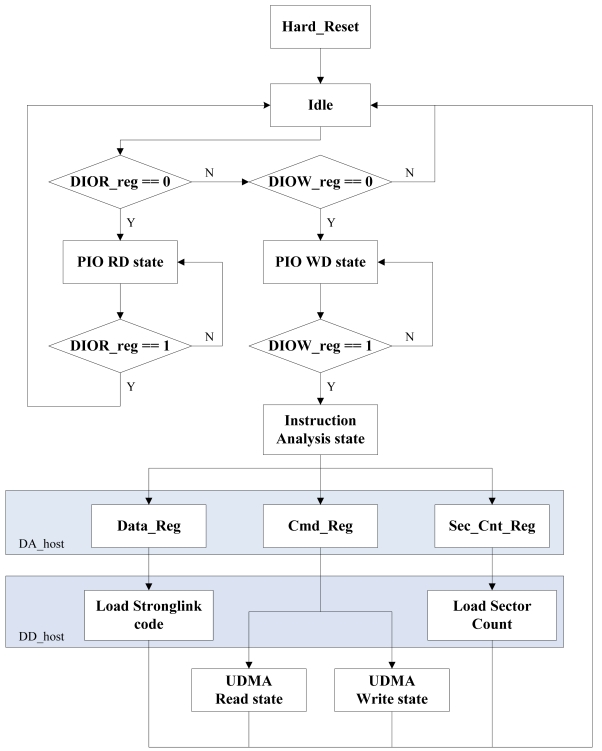
The flowchart of the instruction analysis.

**Figure 9.**
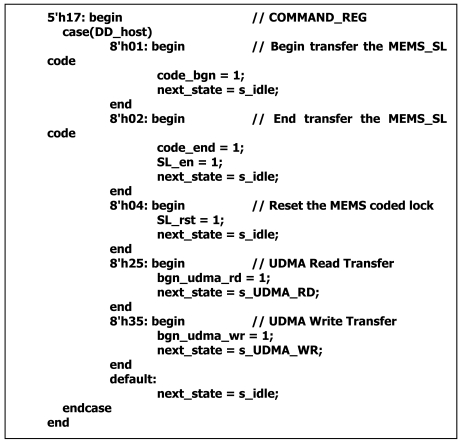
Key Code of the instructions analysis.

The ATA protocol command decoder module (see Figure 7) is the master control unit, which manages the data encryption/decryption module, the MEMS coded lock control circuit module and so on. Figure 8 illustrates the flowchart of the instruction analysis. The main instruction analysis program is shown in Figure 9. In Figure 7, the signals with HOST suffix denote the communication between the ATA protocol command decoder module and the host computer. The signals with DEVICE suffix denote the communication between the ATA protocol command decoder module and the hard-disk. In the module, only data bus is bidirectional. In addition, the left signals of the module are input signals and the right signals are output signals.

According to the command register value, the command decoder module creates the UDMA channel, the PIO channel and MEMS coded lock control channel. The data through the UDMA channel is encrypted or decrypted by AES arithmetic. The data through the PIO channel is directly transferred. Through the MEMS coded lock control channel, the driving and reset instructions of MEMS coded lock are transferred. Except for these command register values, such as 08H (DEVICE RESET), ECH (IDENTIFY DEVICE), 25H (READ DMA EXT) and 35H (WRITE DMA EXT), the reserved 01H, 02H and 04H are used to express the driving start, the driving end and the reset instructions of the MEMS coded lock, respectively (Figure 9).

## 5. The Data Hardware Encryption/Decryption

### 5.1. The Framework of the Data Encryption/Decryption Module

**Figure 10.**
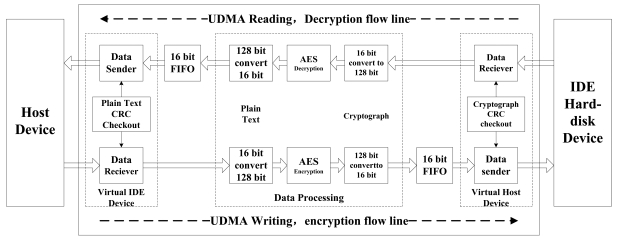
The framework of the data encryption/decryption module.

The data encryption/decryption module (see Figure 1) is indicated in Figure 10, which consists of the virtual IDE device, the data processing module and the virtual host device. There are two data flow lines in Figure 10, named as the decryption flow line and the encryption flow line.

#### 5.1.1. The decryption flow line

The decryption flow line is composed of the data receiver module, the module to convert the 16 bit width data to the 128 bit width data, the AES decryption module, the module to covert the 128 bit width data to the 16 bit width data, the 16 bit width FIFO module and the data sender module.

The cryptograph data from the hard disk is decrypted and transferred to the host device. The working flow of the decryption flow line is as follow: (1) The cryptograph data on the hard disk is received by the data receiver module; (2) The 16-bit width cryptograph data is converted into the 128-bit width cryptograph data; (3) The 128-bit width cryptograph data is decrypted into the plain text by the AES encryption module; (4) The 128-bit plain text is converted to the 16-bit width plain text; (5) The 16-bit width plain text is sent to the 16-bit width FIFO module; (6) The data sender module in virtual IDE device sends the plain text to the host computer.

The cryptograph CRC checkout module (see Figure 10) will compute the CRC value of the data from the hard disk. Then the hard disk will compare the calculated CRC value in the cryptograph CRC checkout module with the calculated value in its own CRC calculation, and will affirm that whether the data is transferred correctly or not.

After the data is sent by the data sender module, the plain text CRC checkout module (Figure 10) will compare the calculated CRC value in the host device with that in the plain text CRC checkout module, and will verify whether the data is transferred correctly or not. If both of the CRC Checksums are right, the data transfer is correct.

**Figure 11.**
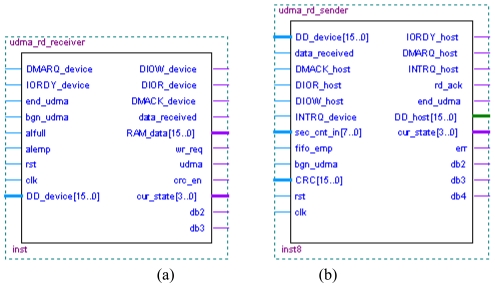
(a) RD_Receiver module. (b) RD_Sender module.

The data receiver module is named as UDMA_RD_Receiver [Figure 11(a)], which simulates the host device and communicates with the hard-disk. The data sender module is named as UDMA_RD_Sender (Figure 11b), which simulates the hard-disk, and communicates with the host device.

#### 5.1.2. The encryption flow line

The encryption flow line is composed of the data receiver module, the module to convert the 16-bit width data to the 128-bit width data, the AES encryption module, the module to convert the 128-bit width data to the 16-bit width data, the 16-bit width FIFO module and the data sender module.

The plain text data from the host device is encrypted and transferred to the hard disk. The encryption flow line is as follows: (1) The plain text data from the host device is received by the data receiver module; (2) The 16-bit width plain text is converted to the 128-bit width plain text; (3) The 128-bit width plain text is encrypted to the cryptograph by the AES encryption module; (4) The 128-bit cryptograph is converted to the 16-bit width cryptograph; (5) The 16-bit width cryptograph is sent to the 16-bit width FIFO module; (6) The data sender module receives the cryptograph from the FIFO module and sends the cryptograph to the hard disk.

After the data receiver module receives the data from the host device, the plain text CRC checkout module will compare the calculated CRC value in the host device with that in its own CRC calculation and will affirm that whether the data is transferred correctly or not. After the data is sent by the data sender module, the plain text CRC module will calculate the CRC value of the transfer data. The hard disk then will compare the calculated CRC value in the data sender module with its own calculated CRC value and will verify whether the data is transferred correctly or not. If both of the CRC Checksums are right, the data transfer is correct.

**Figure 12.**
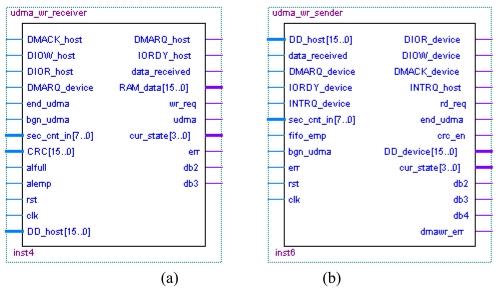
(a) WR_Receiver module. (b) WR_Sender module.

The data receiver module is named as the UDMA_WR_Receiver [Figure 12(a)], which simulates the hard-disk and communicates with the host device. The data sender module is named as the UDMA_WR_Sender [Figure 12(b)], which simulates the host device and communicates with the hard-disk.

### 5.2. The AES Encryption Module and the AES Decryption Module

**Figure 13.**
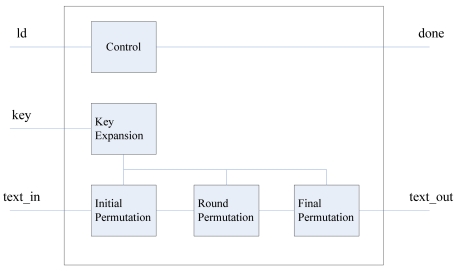
Diagram of AES encryption Module.

The AES encryption module (see Figure 10) is depicted in Figure 13, which includes the key expansion unit, the initial permutation unit, the round permutation unit and the final permutation unit. When ld signal is valid, the encryption module begins to read the key and plain text. After the encryption operation is done, the done signal is kept valid for one clock cycle.

**Figure 14.**
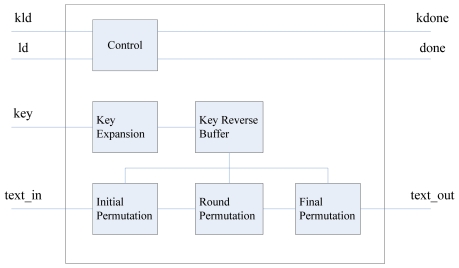
Diagram of AES decryption Module.

The AES decryption module(see Figure 10) is depicted in Figure 14, which includes the key expansion unit, the key reverse buffer unit, the initial permutation unit, the round permutation unit and the final permutation unit. If the kld signal is valid, the decryption module will read the key. Then the expanded key is sent to the key reverse buffer. When the key expansion operation is done, kdone signal is set and kept valid for one clock cycle. After that, the done signal is kept valid for one clock cycle.

### 5.3. The Data Format Conversion and the CRC Check

**Figure 15.**
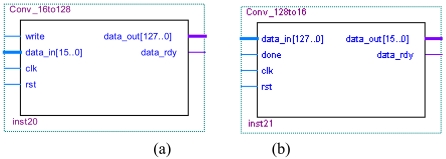
(a) Convert 16bits to 128bits. (b) Convert 128bits to 16bits.

The AES data width is 128 bit, and the data width of the virtual host divice/the virtual IDE device is 16 bit. The data converting module between 128 bit and 16 bit is needed. In Figure 15(a), the 16 bit width data is converted to the 128-bit width data. In Figure 15(b), the 128-bit width data is converted to the 16 bit width data.

**Figure 16.**
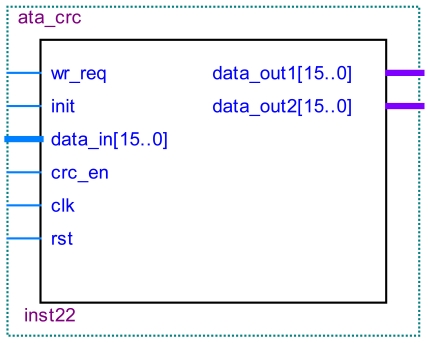
CRC module.

The plain text CRC checkout module and the cryptograph CRC checkout module have the same structure. Their details are shown in Figure 16. The plain text CRC module is located in the virtual IDE device. The cryptograph CRC module is located in the virtual host device. Both of them will be initialized with a seed of 4ABAh at the beginning of an Ultra DMA burst. At the end of the Ultra DMA burst, the host device(or the virtual host device) will send the CRC calculation results to the hard-disk (or the virtual IDE device). If the CRC data in the host device(or the virtual host device) does not match with the calculated value in its own CRC calculation, the hard-disk (or the virtual IDE device) will report that the error has occurred.

### 5.4. The 16 Bit Width FIFO Module

**Figure 17.**
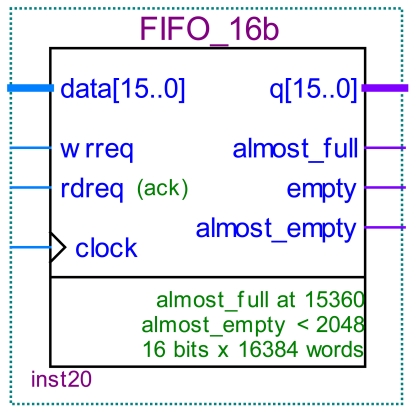
FIFO module.

The 16 bit width FIFO module (Figure 10) is indicated in Figure 17. The module controls the data transfer by the empty pin, the almost_full pin and almost_empty pin. When the memory capacity of FIFO is nearly full, the almost_full pin will inform the host device to pause the UDMA transfer. When the FIFO memory capacity is nearly null, the almost_empty pin will inform the host device to resume the UDMA transfer. When the FIFO memory capacity is empty, the empty pin will inform the host to send the data or finish the UDMA transfer.

### 5.5. The Simulation of the UDMA Data Transfer

**Figure 18.**
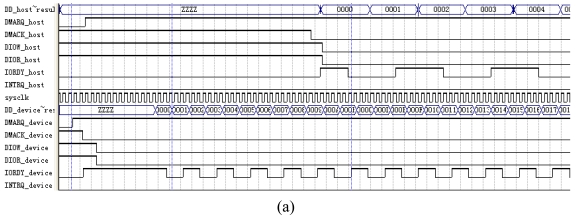

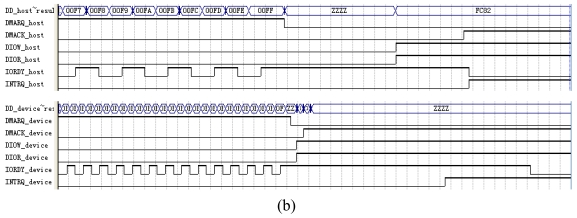
(a)Simulation of initiating an UDMA data-in burst. (b) simulation of device terminating an UDMA data-in burst.

As shown in Figure 18, after the DMARQ_device signal is asserted by the hard disk, the DMACK_device signal, the DIOW_device signal and the DIOR_device signal are set to ‘0’ by the virtual host device. At the same time, the virtual IDE device asserts the DMARQ_host signal. When IORDY_device signal changes, the data is transferred to the virtual host device. Then the data is decrypted by the AES decryption module. When the first decryption word reaches the virtual IDE device, the virtual IDE device generates the IORDY_host signal. When IORDY_host signal changes, the plain text is sent to the host computer in turn.

After the hard disk transfers the last word, the DMARQ_device signal is set to invalidation, the DIOW_device signal is set to validation, and DIOR_device signal is set to invalidation. Then the virtual host device sets the DMACK_device signal invalid, and sends the CRC calculation result to the hard disk. The hard disk compares the CRC calculation result in the virtual host device with the hard disk's CRC calculation value.

After the virtual IDE device transfers the last word, the DMARQ_host signal is set to invalidation. The virtual host device sets the DIOW_device signal valid, and sets the DIOR_device signal invalid. Then the host device sets the DMACK_host signal invalid. The virtual IDE device latches the CRC value from the host device and compares the CRC value in the host device with its own plain text CRC calculation result. When the CRC calculation result in the virtual host device agrees with the hard disk's CRC calculation value, and the host device's CRC calculation value agrees with the virtual IDE device, the INTRQ_host is set valid to inform the host device that the UDMA data transfer is correct.

**Figure 19.**
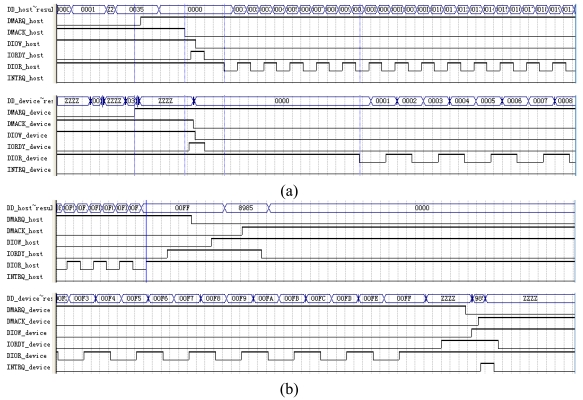
(a)Simulation of initiating an UDMA data-out burst. (b)imulation of device terminating an UDMA data-out burst.

As shown in Figure 19, when the DMARQ_device signal is asserted by the hard disk, the DMARQ_host signal is also asserted by the virtual IDE device. If the DMACK_host signal is valid and the DIOW_host signal is invalid, the IORDY_host signal will be set valid by the virtual IDE device. When the DIOR_device signal changes, the encryption data is sent to the hard-disk in turn. When the host device transfers the last word, the IORDY_host signal and the DMARQ_host signal are set to invalidation by virtual IDE device. Then the host device sets the DIOW_host signal valid. When the host device sets the DMACK_host signal invalid, the virtual IDE device compares the CRC value in the host device with its own CRC calculation value. If both of the IORDY_device signal and the DMARQ_device signal from the hard disk are valid after the virtual host device transfers the last word, the virtual host device makes DIOW_device signal valid and the DMACK_device signal invalid. If the CRC calculation value in the hard disk agrees with the CRC calculation value in the virtual host device and the CRC calculation value in the virtual IDE device agrees with the CRC calculation value in the host device, the INTRQ_host is set valid to inform the host device that the data transfer is correct.

## 6. The MEMS Coded Lock and Its FPGA Circuit

The MEMS coded lock [9] is a kind of the switch mechanism used in the high consequence system. The three safety themes for high sequence systems are isolaton, incompatibility, and inoperability [10]. MEMS coded lock plays an important role in these themes. Sandia National Laboratories in USA has designed a kind of MEMS coded lock by surface microfabrication technology [11-13]. Our MEMS coded lock is fabricated by LIGA-like process. It can store the key in its mechanical structure, realize the authentication function and provides the cipher key to the AES encryption/decryption module. The reset mechanism can resume the MEMS coded lock when MEMS coded lock is locked up. Two photo-electricity couplers provide the mechanical key to the AES encryption module.

### 6.1. The Configuration of MEMS Coded Lock

**Figure 20.**
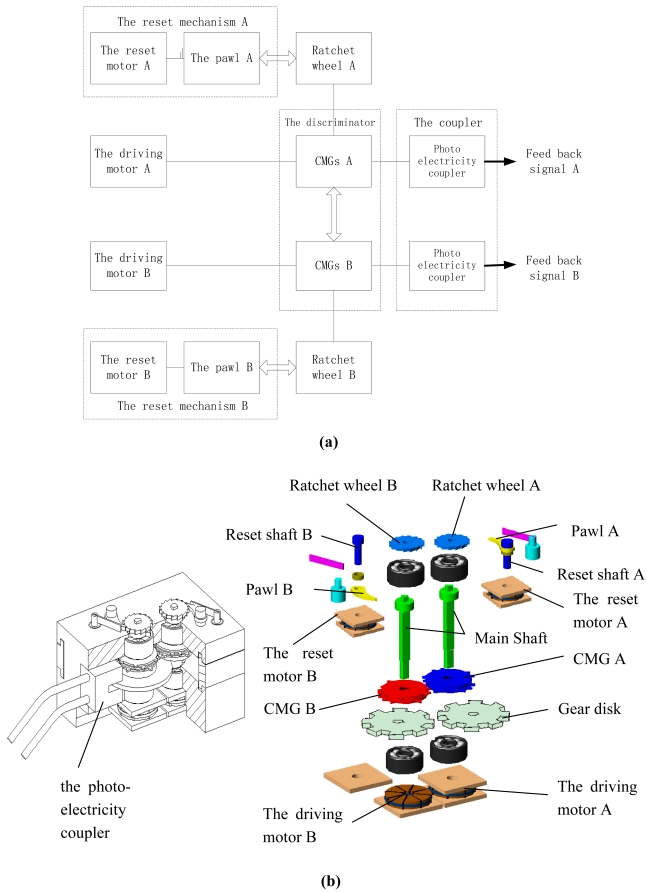
The configuration of the MEMS coded lock: (a) System relationship; (b) Cutaway view and explosion diagram.

**Figure 21.**
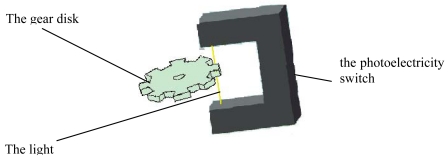
The coupler.

As shown in Figure 20, the MEMS coded lock is composed of the drivers, the discriminator (two groups of CMGs), the reset mechanism (the reset micromotor A/B and the pawl A/B) and the photo-electricity couplers (which composists of one gear disk and one photoelectric switch, Figure 21). The rotor of the driving micromotor, the gear disk, ratchet wheel and a group of CMGs of the discriminator are installed on the main shaft to avoid the transmissional element. And the rotor of the reset micromotor and the pawl are installed on the reset shaft.

### 6.2. The Operation Principle of the MEMS Coded Lock

**Figure 22.**
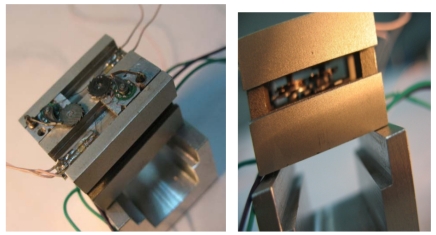
Photos of the MEMS coded lock.

When the password is received by the MEMS coded lock, the driving motor A/B drives CMG A/B running to a specified position. When the user's password is right, two groups of CMGs keep away from contacting with each other. The correct password drives the CMGs to the right position. Then the photoelectricity coupler is opened and the feedback signal is sent. When the user's password is error, the MEMS coded lock is locked and needs to be reset. When the system is being reset, the reset motor puts up the pawl, and the corresponding group of CMGs returns to the right position. The photo of the MEMS coded lock is shown in Figure 22.

### 6.3. The Drivers

**Figure 23.**
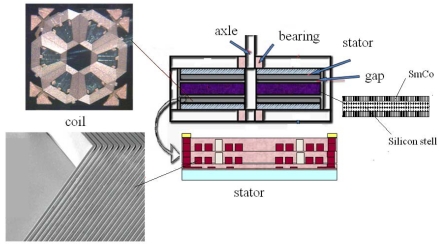
The configuration of the motor.

The drivers are axial electromagnetic motors. In 2001, our unit has reported an axial flux electromagnetic micromotor [14]. To applied the micromotor to the MEMS coded lock, we choose two groups of motors with different dimensions: the driving motors and the reset motors. The driving motor is used to drive the discriminator. The reset motor is used to drive the reset mechnism. The configuration of the motor is shown in Figure 23. The layers of the stator coils are modified to two layers. The rotor is composed of two layer SmCo alloy and silicon steel sheet (Figure 23). The exterior dimensions of the driving motors and reset motors are 6.7 × 6.7 mm^2^, 4.9 × 4.9 mm^2^, respectively.

### 6.4. The Discriminator

The discriminator is composed of two groups of CMGs and two groups of the ratchet wheels and pawls. Each group of CMGs is composed of multi-level gears driven by their own motor,and the level number of gears is equivalent. The two groups of CMGs rotate in the same direction. The code is solidifled by setting teeth on differential gears, and discriminated by the given rotation of the two groups of CMGs.

**Figure 24.**
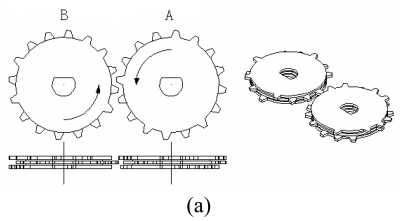

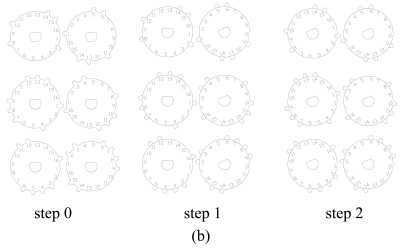
The structure of the CMGs: (a) the configuration of the mechanism, (b) The discrimination process.

Table 1 shows the parameters of the rathcet and pawl mechanism. The two groups of CMGs corresponding to the code sequence“ABAABABBBBAABBBAAABAAABBAABBABAB”, is illustrated in Figure 24. The two groups of CMGs rotate in anticlockwise direction [Figure 24(a)]. In step 0, CMG A and CMG B are the initial state. In step 1, “A” in the code sequence is received, CMG A rotates a step (22.5°) in the anticlockwise direction, and CMG A and CMG B pass through without interfere. In step 2, if the right code “B” is received, the CMG B rotates a step (22.5°) in the anticlockwise direction, and CMG A and CMG B pass through without interfere. Otherwise, in step 2, if the wrong code “A” is received, CMG A rotates in the anticlockwise a step(22.5°), and then the the teeth in the middle layer of the CMG B and CMG A will interfere in the next step. Thus two groups of CMGs not only rotate in anticlockwise direction, but also rotate in reverse (the ratchet wheels and pawls can hold CMGs to rotate in the anticlockwise direction), the discriminator will be locked.

### 6.5. The Coupler

As shown in Figure 21, the coupler includes the photoelectricity switch and gear disk. When the correct code is received, the gear disk is driven by the driving motor. The the notch of the gear disk is the position, where the ligth can passthrough. Then through the photoelectricity conversion, the electronical signal is sent.

### 6.6. The Main Process

#### 6.6.1. The micromaching process of the electromagnetic motor's stator's coils

**Figure 25.**
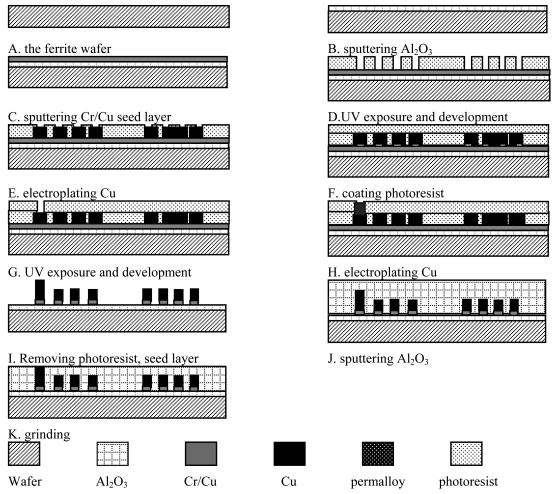
The micromaching flowchart of the motor's coils.

In [15], the electrostatic actuator has been constructed using a multi-level, LIGA-like process. In our work, the two-layered coils of the electromagnetic motor's stators are also fabricated by an similar multi-leve process. Figure 25 is the flowchart of the micromaching process. After the step K, the second layer coil begins to been fabricated. The second layer coil's micromaching process is similar to the first layer coil' micromaching process, so we only give the process flowchart of the first layer coil.

#### 6.6.2. The machinging process of the electromagnetic motor's rotor

**Figure 26.**
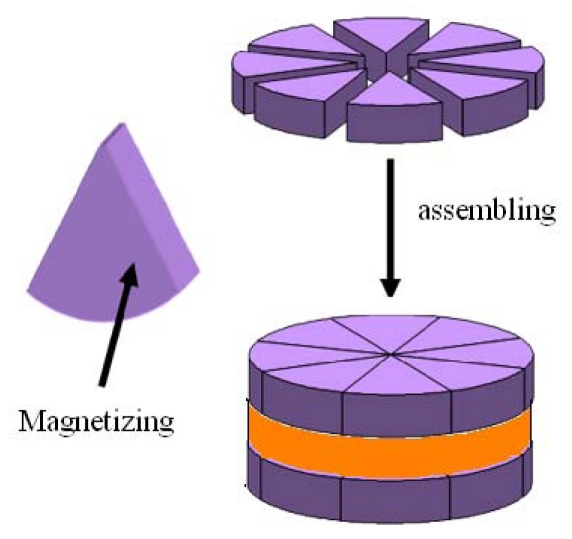
the maching process of the rotor.

WEDM(Wire Electrical Discharge Machining) has been used to fabricate microstructure [16]. In this paper, the permanent material of the rotor is SmCo alloy(Sm 25.5%, Co 52.5%, Fe 15%, Zr 3%). SmCo sectors are cuted by Wire Electrical Discharge Machining. Then these sectors are Magnetized and assembled on the circular silicon stell sheet (Figure 26).

#### 6.6.3. The micromaching process of the Ratchet wheel, pawl and CMG

**Figure 27.**
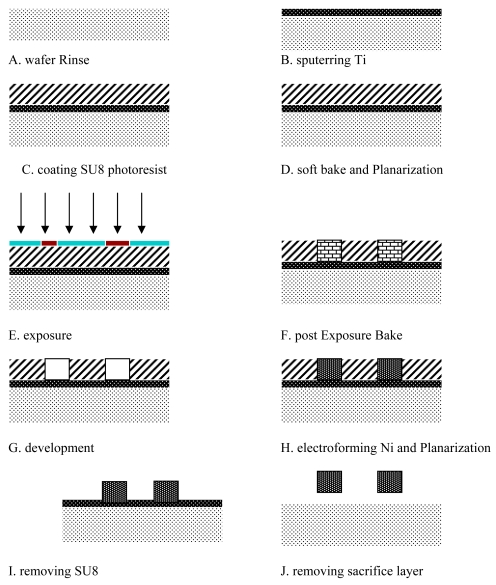
The micromaching process of the Ratchet wheel, pawl and CMGs.

The Ratchet wheel, pawl and CMGs are fabricated by an LIGA-like process based on SU-8 [17-19]. The main microfabrication flowchart is shown in Figure 27.

#### 6.4. The MEMS Coded Lock Control Circuit Module

**Figure 28.**
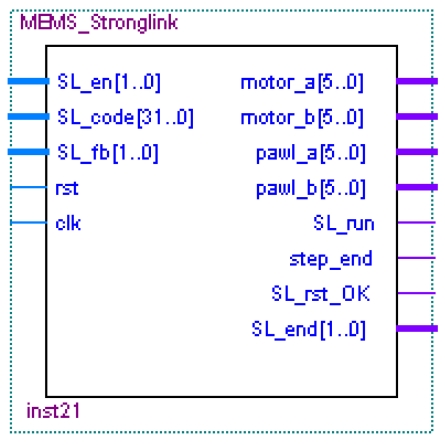
MEMS strong-link controlling circuit module.

The MEMS coded lock circuit module (Figure 28) receives the user's password from the ATA command decoder module by the SL_code pins. Then the module drives the MEMS coded lock according to discriminate user's password. If MEMS coded lock is locked, the module will send the error signal to the host device by the ATA protocol command decoder module.

When SL_en signal is valid, the signals from SL_code pins are used as the driving series of the MEMS coded lock, and the SL_run pin of the module informs the cipher key management module to receive the feedback signals of the MEMS coded lock. At the same time, the module compares the feedback signals of the MEMS coded lock with the user's password to affirm whether the driving operation of the MEMS coded lock is finished normally or not.

After the MEMS coded lock's running action is finished, the running result of the MEMS coded lock is sent to the ATA command decoder module by SL_end pin. Then the ATA command decoder module sends the result to the host device. If the two groups of CMGs in MEMS coded lock are locked up, the module will drive MEMS coded lock to the correct status. If the reset operation of the MEMS coded lock is successful, the signal sent by SL_rst_OK pin will inform the ATA protocol command decoder module the status of the MEMS coded lock.

#### 6.5. The Cipher Key Management Module

**Figure 29.**
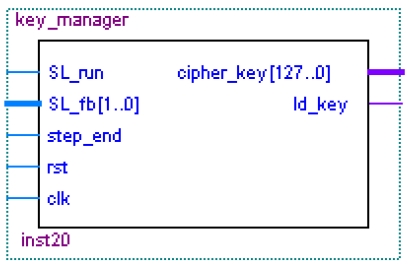
Key management module.

The cipher key management module (Figure 29) produces the 128-bit cipher key according to the feedback signals of the MEMS coded lock. If SL_run signal is valid, the cipher management module changes the status from the idle status to the working status and begins to run. If the step_end signal is valid, the cipher management module knows that MEMS coded lock has rotated a big step. SL_fb signal from the MEMS coded lock is used to generate the encryption/decryption key. The signals from Cipher_key pins will provide 128 bit key for the encryption/decryption module. After the cipher key is generated, ld_key tells the encryption/decryption module to load the key.

After the discrimination micromotors run a step, the corresponding switch signal (off or on) of the coupler is outputted to the key management module. When discriminating operation is correct, two groups of CMGs alternately rotate 32 steps. After each step movement is finished, 2 bit feedback signal is outputted. Thus the cipher key management module totally received 64 bit feedback signals. After that, the key management module combines 64 bit feedback signals to 128 bit key, which will be sent to the encryption/decryption module.

#### 6.6. The Cipher Key Generation Mechanism

**Figure 30.**
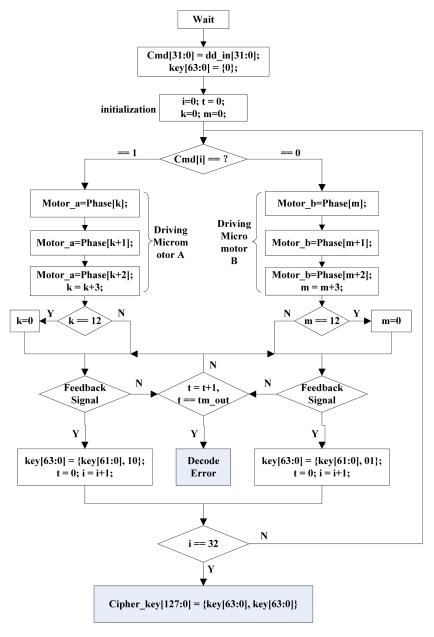
Flowchart of the cipher key generation.

Figure 30 is the flowchart of the cipher key generation, which denotes the generation process of the 128 bit key. User's input password is used as the discrimination sequences to drive the MEMS lock to decode. Every bit of user's password is inquired. If the current bit is 1, the micromotor A rotates a step. If the current bit is 0, the micromotor B rotates a step. When MEMS coded lock rotates a step, the cipher key management module waits for receiving the feedback signal from the MEMS coded lock. The decoding action is error, if the cipher management module can not receive the feedback signal after a certain time. If the cipher management module can receive the feedback signal after a certain time,the change of feedback signals is used to fill the bits of the cipher key. Finnally, the 64-bit cipher key is multiplexed to the 128-bit cipher key.

#### 6.7. The Simulated Test of the MEMS Coded Lock Controlling Circuit

**Figure 31.**
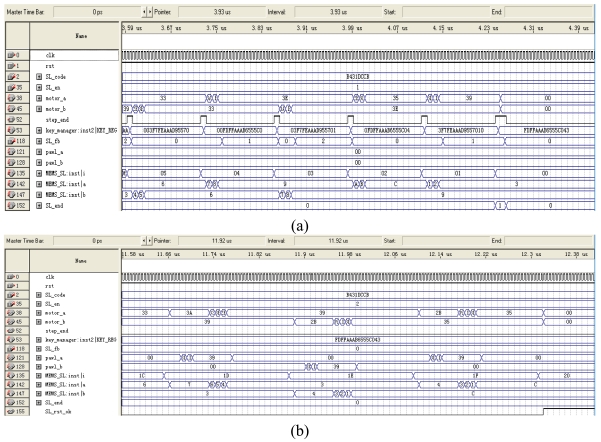
(a)Simulated waveform of MEMS coded lock's discrimination. (b)Simulated waveform of MEMS coded lock's reset.

The program for controlling MEMS coded lock and generating the cipher key is coded by the Verilog HDL language. The syntheses and simulation are done by Quartus II software [20]. The simulated waveform of MEMS coded lock's discrimination and reset operation are shown in Figure 31.

In the simulated waveform of Figure 31, all signals' values are hexadecimal. The basic clock frequency is 50 MHz (clk signal). When SL_en signal is 1, the MEMS coded lock begins to decode according to the value of the SL_code. When SL_en signal is 2, MEMS coded lock begins to reset.

After every bit of the user's password is discriminated, the step_end signal of the MEMS coded lock controlling circuit module will be sent to the cipher key management module. Then the received SL_fb signals are combined into 64 bit expansion key. If the decoding processing is error, SL_end signal is equal to 1, which informs the ATA protocol command decoder module that the MEMS coded lock's decoding action is error.

During the resetting process, the ratchet wheel steps a small phase in the positive direction. Moreover, the pawl steps 3 small phases and stops. Afterward, the ratchet wheel steps 4 small phases in the reverse direction and stops. Finally, the pawl_a or the pawl_b signal of the MEMS coded lock module is set to ‘0’, and the pawls are resumed to the original position because of the leaf spring's force. Thus, the ratchet wheel is locked by the pawl. During the reset processing, the step_end signal is kept ‘0’. If the reset processing is right, SL_rst_OK is valid, which informs the ATA protocol command decoder module that the reset of MEMS coded lock is successful.

During the running process of the MEMS coded lock, motor_a and motor_b signals are valid, which control one of the two groups of CMGs to keep on the special phase position. When the MEMS coded lock is in the status of the single step reset, the pawl_a and pawl_b signals are valid, which makes the pawl put up and the ratchet wheel rotate reversely. The pawl locks the ratchet wheel between the two single step reset status, and the ratchet wheel can not change the current phase position.

After the user's password is discriminated by the MEMS coded lock, ld_key signal will be valid, which denotes that the 128 bit cipher key has been generated. The encryption/decryption module immediately loads the cipher key.

## 7. Test of the Portable Hard Disk Encryption/Decryption System

**Figure 32.**
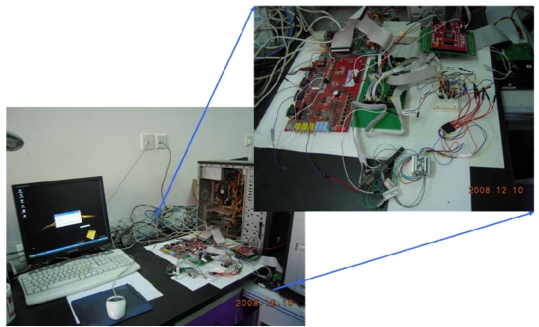
The prototype of the portable hard disk encryption/decryption system with MEMS coded lock.

As shown in Figure 32, the prototype of the portable hard-disk encryption system includes GIGABYTE (P4V800D-X) main board,SEAGATE 160G hard disk and Windows XP.

### 7.1. The Function Test

**Figure 33.**
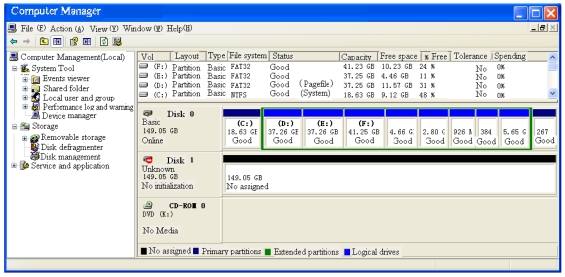
Read encrypted hard disk without the portable hard-disk encryption/decryption system.

**Figure 34.**
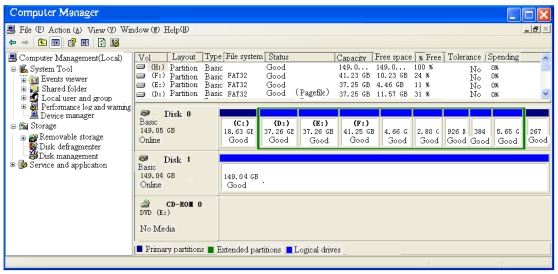
Read encryted hard disk with the portable hard-disk encryption/decryption system.

After the host computer is powered on, the USB control program is downloaded to the USB portable hard-disk interface card by Keil C702 tool [21]. Then, the VHDL program is downloaded to the FPGA portable hard-disk data encryption/decryption card by QuartusII tools [20]. Using the microscope, MEMS coded lock is adjusted to the original status. In succession, the USB portable hard-disk interface card, the FPGA portable hard-disk data encryption/decryption card, MEMS coded lock and the hard disk are linked to the portable hard disk encryption system by the cable. The functional test is done as follows:

1. The portable hard disk encryption system is inserted into the host computer. Then, the hard disk is formatted after the correct password is inputted. Finally, the host computer will store the data file into the hard disk.
2. The host computer could not identify the hard disk without the portable hard-disk encryption system (Figure 33). The host computer regards the hard disk as an unformatted hard disk. In this case, the host computer could not read the encryption data in hard disk, and the identifiers could not be found in the explorer of the host computer.
3. If the hard disk is linked to the host computer with the portable hard disk encryption system, the host computer could get the basic information of the hard disk of Figure 34). Then the hard disk would appear in the explorer, and the data of the hard disk could be read/written.

The function test showed that only when the authentication of user's password is passed, the user could use the hard disk, and the hard disk can appear in the explorer of the host computer. Illegal user could not read and write the hard disk, if he has no the portable hard-disk encryption/decryption system and authentication password.

### 7.2. The Transmission Speed Test

**Figure 35.**
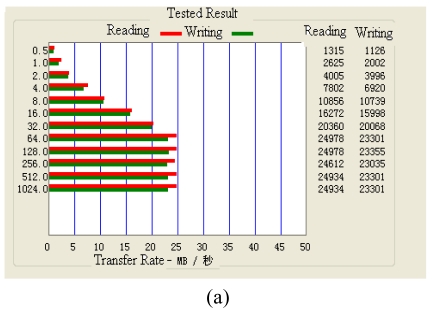

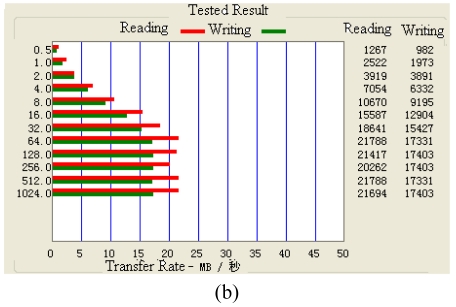
(a) Result without encryption systemand. (b) Result with encryption system.

The ATTO disk Benchmark [22] is used in the data transmission speed test experiment. First, we prepared the files in the host computer's hard disk. In succession, we insert the portable hard disk encryption/decryption system to the host computer's USB interface. And the ATTO disk Benchmark is run. Then we copy the test files from the hard disk of the host computer to the tested hard disk. The read/write speed of the hard disk is tested according to different packages sizes, which vary from 0.5KB, 1.0KB, 2.0KB, and till to 1024KB. The test of the read/write speed of the 160G hard disk is shown in Figure 35. If the package size is less than or equal to 64kb, the read/write speed of the hard disk will increase as the package size increasing (Figure 35). When the package size is greater than 64KB, the read/write speed of the hard disk will not increase anymore (Figure 35). Finally, the data reading and writing speed of the hard disk without the portable hard disk encryption/decryption system are 23MB/s and 24MB/s, respectively. The data reading and writing speed of the hard disk with the portable hard disk encryption/decryption system are 17MB/s and 21MB/s, respectively.

## 8. Conclusion

In this paper, the portable hard-disk encryption/decryption system is developed. The key for the authentication module and the encryption/decryption module is generated by MEMS coded lock. This is a kind of novel method to keep data safe by MEMS structure. The hard disk interface controller, the encryption/decryption circuit, MEMS coded lock's controlling circuit and the cipher key management circuit are realized by FPGA. The USB portable hard-disk interface card is realized by the Cypress chip. Finally, the prototype is fabricated and tested successfully.

## Figures and Tables

**Table 1.** The parameters of the ratchet wheel and pawl mechanism.

**The addendum circle diameter**	**The tooth depth**	**root circle diameter**	**The angle of the dental socket**	**The number of the teeth**	**the modulus**	**The length of the pawl**
**4.8 mm**	0.3 mm	4.2 mm	60 °	16	0.3 mm	4.0 mm
